# Calcium Oscillatory Behavior and Its Possible Role during Wound Healing in Bovine Corneal Endothelial Cells in Culture

**DOI:** 10.1155/2019/8647121

**Published:** 2019-02-21

**Authors:** Cristian Justet, Silvia Chifflet, Julio A. Hernandez

**Affiliations:** ^1^Departamento de Bioquímica, Facultad de Medicina, Universidad de la República, Gral. Flores 2125, 11800 Montevideo, Uruguay; ^2^Sección Biofísica, Facultad de Ciencias, Universidad de la República, Iguá s/n esq. Mataojo, 11400 Montevideo, Uruguay

## Abstract

In epithelial layers in culture, immediately after an injury a fast calcium wave (FCW) propagates from the wound borders toward the rest of the monolayer. We show here that similarly to other tissues, during the FCW in bovine corneal endothelial (BCE) cells in culture many cells exhibit calcium oscillations mediated by IP3 signaling. In this study we perform a detailed characterization of this oscillatory behavior and explore its possible role in the process of wound healing. In previous work we showed that, in BCE cells in culture, the healing cells undergo two stages of caspase-dependent apoptosis, at approximately two and eight hours after wounding. We determined that inhibition of the FCW greatly increases the apoptotic rate of the two stages, suggesting that the wave prevents excessive apoptosis of the healing cells. Taking this into account, we investigated the possible participation of the calcium oscillations during the FCW in apoptosis of the healing cells. For this, we employed ARL-67156 (ARL), a weak competitive inhibitor of ecto-ATPases, and the calcium chelator EGTA. We show here that, in healing BCE cells, ARL enhances cellular calcium oscillations during the FCW, while EGTA decreases oscillations. We found that ARL produces a significant decrease (to about half the control value) in the apoptotic index of the first stage of apoptosis, while EGTA increases it. Neither drug noticeably affects the second stage. We have interpreted the effect of ARL on apoptosis as due to the maintenance of moderately risen ATP levels during the FCW, which is in turn the cause for the enhancement of ATP-dependent calcium oscillations. Correspondingly, EGTA would increase the apoptotic index of the first stage by promoting a decrease in the calcium oscillatory rate. The fact that the second stage of apoptosis is not affected by the drugs suggests that the two stages are at least partially subject to different signaling pathways.

## 1. Introduction

Wound healing is a property of the utmost importance for organisms. Even under culture conditions, most cellular layers exhibit the capacity to restitute injured areas by migration and proliferation of the border cells. Due to their characteristic functions and localizations, epithelial tissues have been particularly employed as models for the study of the basic aspects of the healing processes, both under in vitro and in vivo conditions. In essence, an experimental wound produced on an epithelial monolayer in culture determines a collective cellular response that, although more conspicuous at the wound borders, nevertheless also involves cells located several rows away [1–3]. Depending on the healing mode, the cells undergo dramatic morphological modifications and perform cohesive and/or individual migration into the denuded area [4, 5]. Later in the healing process, proliferation of the migrating cells contributes to tissue restitution to an extent that depends on the particular cell type [6, 7]. Many cells undergo apoptosis, a process that has been shown to be involved in the control of the migration and proliferation rates of the healing cells [7–10].

Wound healing is triggered and regulated by mechanisms still incompletely understood. In most epithelia, a fast calcium wave (FCW) occurs immediately after the production of a wound, propagating from the border cells toward the rest of the monolayer with a typical duration of several minutes [7, 11–16]. Some epithelia also exhibit another calcium wave that starts to develop approximately one hour after healing and has a slower rate of spreading into the monolayer (“slow calcium wave” [16]). Other phenomena that take place as early consequences of an injury are the establishment of a hydrogen peroxide gradient [17, 18] and the development of ERK1/2 waves [19]. In particular, the FCW has been suggested to signal different cellular responses that participate in the healing process [6, 12, 18, 20, 21]. Employing a model of mechanical injury in bovine corneal endothelial (BCE) cells in culture, we have shown that the FCW is also involved in the control of the apoptotic response of the healing cells [7]. The increase in extracellular ATP that occurs as a consequence of its release from the damaged cells and by mechanical stimulation of the surviving border cells seems to represent the main signal to trigger the FCW [22–24].

Calcium signaling participates in the regulation of a myriad of physiological processes, such as cell proliferation, migration, survival, and apoptosis [25–28]. Many types of dynamic responses of cellular calcium to diverse stimuli have been observed, from simple transients to more complex behaviors, such as oscillations and even chaos [29]. These behaviors have been interpreted with the aid of mathematical models of varied complexity [30]. For the case of the FCW triggered by a mechanical tissue injury, many cells exhibit oscillations in cytosolic calcium [31–34]. This has also been observed by mechanical stimulation of uninjured monolayers, a procedure that provokes a calcium response similar to the FCW [35–37]. In human urothelial cells in culture, the cells exhibit a very large diversity of dynamic behaviors of the calcium modifications, a property that has been suggested to underlie the vast and complex signaling processes involved in the overall healing response [33]. For this experimental model, the authors made a thorough characterization of the dynamic responses in cytosolic calcium exhibited by the different cells. In imaginal discs of* Drosophila* larvae the variability in the calcium responses to laser ablation was correlated with cell size and mechanical anisotropy [38]. The dynamic complexity of the calcium signals exhibited by epithelia has also been observed in uninjured tissues and suggested to have a genetic basis [39].

We show here that, in BCE cells in culture, many cells also exhibit an oscillatory behavior during the FCW. It has been suggested that calcium oscillations may in general not solely be a contingent consequence of complex dynamic interactions but have specific physiological roles, where the frequency and amplitude of the oscillations possess informational significance [40–42]. In particular, calcium oscillations have been found to promote mouse oocyte activation [43] or apoptosis [44], depending on the oocyte age. It has also been suggested that calcium oscillations could mediate survival responses in neurons and T-lymphocytes [45]. As stated, ATP plays a major role in triggering calcium signals. In uninjured tissue studies, the nucleotide has also been specifically held responsible for the generation of calcium oscillations [36, 46–51] with a strong dependence of the oscillatory frequency on the ATP concentration [46, 52].

As mentioned above, the emergence of cytosolic calcium oscillations during calcium waves triggered by tissue injuries has already been communicated [31–34]. However, to the best of our knowledge, there are no reports regarding the roles of these oscillations in the healing process. The purpose of the present work has therefore been to perform a detailed characterization of the dynamics of the calcium modifications undergone by the healing BCE cells during the FCW and to study the possible role of oscillations in apoptosis.

## 2. Materials and Methods

### 2.1. Reagents and Solutions

Chemicals and drugs were from Sigma Chemical Co. (Munich, Germany) unless otherwise specified. Control solution (CS) is as follows: 137 mM NaCl, 5.4 mM KCl, 1.02 mM NaH_2_PO_4_, 3.6 mM CaCl_2_, 0.8 mM MgSO_4_, and 10 mM HEPES (4-(2-hydroxyethyl)-1-piperazine ethanesulphonic acid) pH 7.4. Final concentrations of the drugs were as follows: cyclopiazonic acid (CPA, Molecular Probes Inc., Eugene, OR), 10 *μ*M; ARL 67156 (ARL) and U73122, 50 *μ*M; pyridoxalphosphate-6-azophenyl-2′,4′-disulphonic acid (PPADS), 10 *μ*M.

### 2.2. Cell Culture and Wounding Procedure

Bovine eyes, obtained fresh from certified slaughterhouses under veterinary supervision (see Acknowledgments), were processed as described in [53]. In short, corneal endothelium was treated with trypsin (0.25%)/EDTA (0.02%) in Ca^2+^ and Mg^2+^-free PBS for 20-30 min in the tissue culture incubator. The endothelial cells were plated in minimal essential medium (MEM) supplemented with 10% serum, 50 *μ*g/ml gentamicin, 0.25 *μ*g/ml amphotericin B, and 50 *μ*g of total protein/ml of retinal extract and grown on glass coverslips. This study only employed cells from the 1st to the 5th passages that had achieved visual confluence at least five days before the experiments. Linear wounds from side to side of the coverslip were made using a 21-gauge syringe needle [54]. For this, the coverslips were mounted on the experimental chamber, immobilized with tape, and placed under the microscope. The time-lapse photography was initiated and then the wound produced manually at the center of the microscopic field.

### 2.3. Cytosolic Calcium

Modifications in cytosolic calcium were assessed by Fluo-4 fluorescence (Molecular Probes Inc., Eugene, OR). This probe was kept as a 1.7 mM stock solution in DMSO at -20°C and, for the experiments, mixed with 20% pluronic acid (Molecular Probes Inc.) in DMSO, and diluted to a final concentration of 1.7 *μ*M in the corresponding solution. The cells grown on coverslips were incubated in this solution at room temperature (RT) for 15 min. After washing with CS, the coverslips were mounted on a homemade chamber [53] containing CS and 1 *μ*g/ml propidium iodide to reveal dead cells. Wounds were produced under the fluorescent microscope and photographs taken every 1 second employing a Moticam Pro 282B camera and the Moticam Image Advanced 3.2 software. For the studies we employed a direct Nikon 50i epifluorescence microscope (Nikon, Tokyo, Japan) with a 10X Plan-Fluor objective and a B-2A Blue Excitation Filter Block (450-490 nm Excitation Filter, 500 nm Dichromatic Mirror Cut and 515 nm Barrier Filter).

### 2.4. Calcium Dynamics

**Figure 1:**
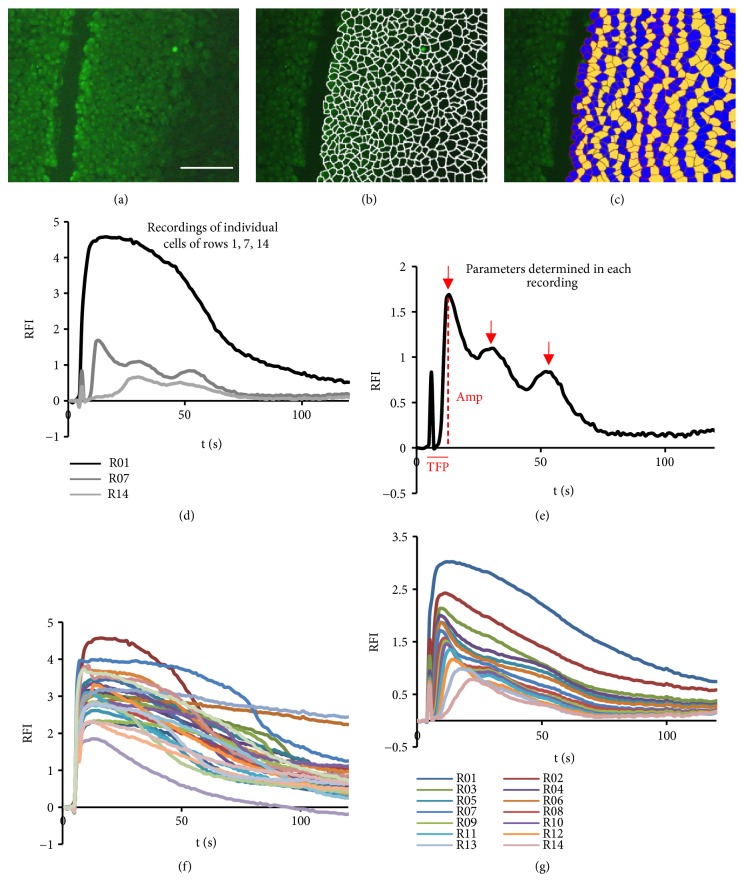
*Characterization of the cellular calcium response during the FCW*. (a-c) Delimitation of the individual cells and classification of the cells into successive rows (see Materials and Methods). (d) Examples of the dynamic behavior of different cells and (e) graphical indication of the signal parameters analyzed in this study. The short peak at the left corresponds to the needle reflection during wounding and sets time zero for the measurements. RFI: relative fluorescence intensity (fold increase over basal value), TFP: time to achieve the maximum fluorescence intensity value of the first peak, and Amp: maximum RFI of the first peak. The arrows indicate the different signal peaks. NP is the total number of peaks in the corresponding trace. For the example shown in (e): row number = 7, TFP = 7s, Amp = 1.69, and NP = 3. We consider the mean cell diameter D to be 35 *μ*m. Therefore V = 35 *μ*m/s (V = row x D/TFP). (f) Dynamic responses of the cells of the first row and (g) average RFI values of the different rows. Bar = 300 *μ*m.

To perform the studies on calcium dynamics, cells were individualized as follows. To better appreciate the cell boundaries, two or three images of the same sequence were arbitrarily selected and merged together (Figure 1(a)). In the resulting image, the limits of each cell were drawn using the Pencil Tool of the Adobe Photoshop Software (Adobe Systems Corporation, San Jose, CA, USA) (Figure 1(b)) and the cells were identified by numbering them with the Count Tool in a row-by-row fashion starting from the wound edge. The first row was defined as the one containing all the cells at the wound border, excluding propidium iodide positive cells. Each following row was defined as the group of cells in contact with the previous row (Figure 1(c)). For each cell, the time course of the calcium signal was then assessed by image analysis of the subsequent micrographs. For this, we created an Adobe Photoshop Action to determine the integrated density of each cell in an ordered fashion according to its number and for each time point of the experiment. Data were then stored in a spreadsheet for analysis. A total of 400 to 500 cells were analyzed per image.

Identification of calcium peaks in the dynamic signals was performed by direct observation of the time plots. Two of us (CJ and JAH) independently classified the cells by counting the number of peaks exhibited by their signals during the two-minute interval following injury. Cells displaying more than one calcium peak were considered to be oscillatory. Comparison of the two independent observations revealed a very high level of agreement. In a few cases, discrepancies were set by a third independent observation (by SC). From the data stored in the spreadsheet (see above) four parameters were derived: the amplitude of the first peak (Amp), the time lapse to reach the first peak after injury (TFP), the number of identifiable peaks (NP), and the wave velocity (V) (Figure 1(e)). The amplitude Amp was measured as the difference between the first maximum of integrated density and the basal value, TFP as the time elapsed between the injury and the first maximum. The calcium wave velocity V was defined as the quotient of the distance from the wound edge (calculated as the number of rows, multiplied by the average diameter of the cells (D)) and the TFP (V = number of rows x D/TFP). The fraction of responding cells was calculated as the number of cells exhibiting one or more calcium peak with respect to the total number of cells in the field (i.e., counted from the wound edge to the end of the field).

In order to evaluate whether the calcium signal jumped over discontinuities, double-wounding experiments were performed. For this, cells were loaded with Fluo-4 AM and two wounds were sequentially produced. The first wound was made on the center of the monolayer. After 10 to 20 min the coverslips were mounted on the chamber with the first wound placed at the center of the field. Then, the second wound was made as close as possible to the first wound (cf.  Figure 3(a)). Single-wound controls were made as usual in independent coverslips.

To provoke cytosolic calcium increase in uninjured BCE monolayers by stimulation with ATP, the coverslips were mounted in the chamber under the fluorescent microscope and a solution of ATP in CS was added to the chamber. In these experiments, images were similarly processed for calcium as above, but data were collected from all the cells contained in a square of 150 *μ*m side drawn at the center of the field.

### 2.5. Calcium Depletion and Inhibition of Ecto-ATPases

To produce calcium depletion of the endoplasmic reticulum, 10 *μ*M CPA was added to the culture medium 30 min before wounding. For experiments performed in the absence of extracellular calcium, cells were incubated in CS without calcium in the presence of 100 *μ*M EGTA one min prior to wounding. To study the effect of ATP-induced calcium oscillations on apoptosis, cells were incubated for 2 min in different ATP concentrations and washed twice with culture medium and 10 *μ*M CPA was incorporated. After 30 min of incubation, the culture medium was removed and the cells were kept for 2 min in Ca^2+^ and Mg^2+^-free PBS with 100 *μ*M EGTA, to achieve FCW abolition [7]. The wounds were performed 30 s after the addition of PBS. The cells were then washed three times with culture medium and maintained for 2 h in the tissue culture incubator.

In order to inhibit ecto-ATPases, 50 *μ*M ARL was added to the medium 10 min before wounding, kept until 5 min after wounding and then the monolayers were washed three times with culture medium.

### 2.6. Apoptotic Cells

In previous work we demonstrated that all the nuclear condensations observed during wound healing in cultured BCE cells are caspase 3 and TUNEL positive and have thus considered them as apoptotic bodies [7]. Therefore, in this study, to identify apoptotic cells the coverslips were fixed with 4% paraformaldehyde (PFA) for 15 min, permeabilized with 0.1% Triton X-100 for 5 min, and incubated with 50 *μ*M Hoechst 33258 for 20 min. The apoptotic index (AI) was determined as the percentage of apoptotic bodies with respect to control produced in the first 20 rows of cells from the wound edge per millimeter of wound border.

### 2.7. Statistical Analysis

The results and images are representative of at least three independent experiments performed in duplicate. Numerical results are expressed as means ± SD. Statistical significance was assessed employing Student's t-test.

## 3. Results

### 3.1. Cellular Calcium Dynamics during the Fast Calcium Wave

Calcium dynamics in the individual cells was studied by processing the image sequences of Fluo-4 signals immediately after wounding, as described in Materials and Methods (Figures 1(a)–1(c)). A movie showing the calcium dynamics during the FCW is included (Supplementary Material, movie 1).  Figure 1(d) shows examples of relative calcium fluorescent signals of three cells, corresponding to rows 1, 7, and 14. Figure 1(e) and its legend describe the signal parameters determined for each case (see Materials and Methods): the amplitude of the first peak (Amp), the time lapse to reach the first peak after injury (TFP), the number of identifiable peaks (NP), and the wave velocity (V). Inspection of the calcium signals displayed by all the cells in each experiment reveals that there is a high range of dynamic behaviors in the cellular responses, from cells that exhibit a maximum and then a monotonous relaxation to basal values to cells that display a clear oscillatory behavior, such as the one shown in Figure 1(e). This variability can be observed for the cells in one row (Figure 1(f)) and persists for the average row tracings (Figure 1(g)). This variability in the behavior of the cellular calcium responses of BCE monolayers is analogous to the one found previously in other tissues for wound-triggered calcium waves [32, 33].

**Figure 2:**
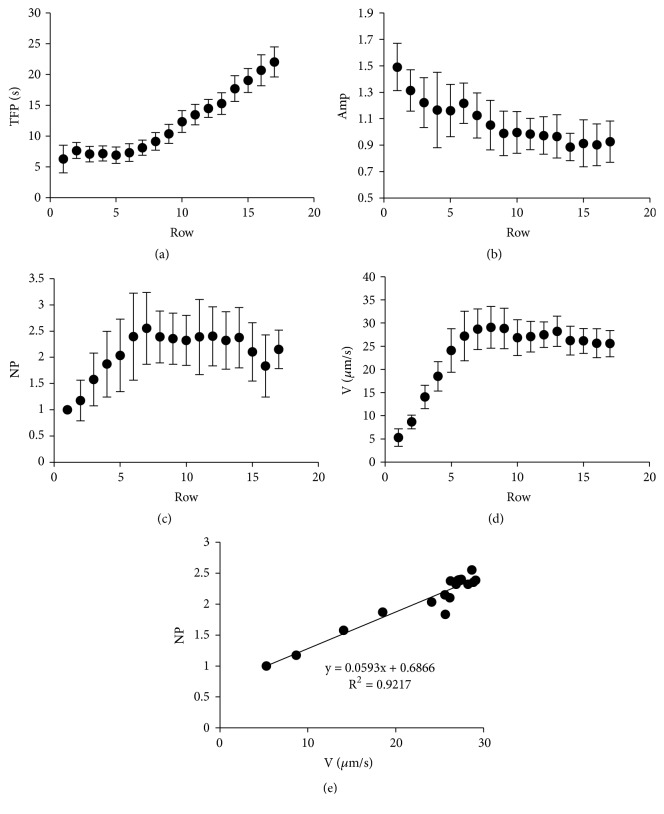
*Average parameter values per row of the dynamic calcium responses under control conditions*. The figure displays the average values and standard deviations for each row of TFP (a), Amp (b), the number of peaks (NP, (c)) and the wave velocity of propagation (V, see legend to Figure 1, (d)). The plot NP vs V (e) reveals a significant linear correlation. The figure shows results obtained from a typical experiment with a total number of analyzed cells equal to 439.

Figure 2 shows the average values obtained for Amp, TFP, NP, and V for each row. As can be seen, the time to reach the first peak increases and the signal amplitude decreases with the row number (Figures 2(a)-2(b)). This is analogous to the results described for wounded urothelial layers [33] and is consistent with the idea that a diffusive signal is involved in the calcium response. Both the number of peaks in each tracing and the wave velocity increase until they reach a maximum between approximately the seventh and the tenth rows (Figures 2(c)-2(d)). A strong linear correlation was found between NP and V (Figure 2(e)), a finding that reveals that both properties simultaneously increase with the distance traveled by the wave. This is consistent with the idea that the calcium oscillations and the wave propagation depend on enhanced IP3 production [39, 55]. A reinforcement of the IP3 signal along the intercellular trajectory of the wave could underlie the increment in the number of peaks and in the wave velocity shown in Figures 2(c)-2(d). In the control experiments, about 94% of the cells exhibited cytosolic calcium increase (cf.  Figure 4). Out of these, 64% showed oscillatory behavior.

**Figure 3:**
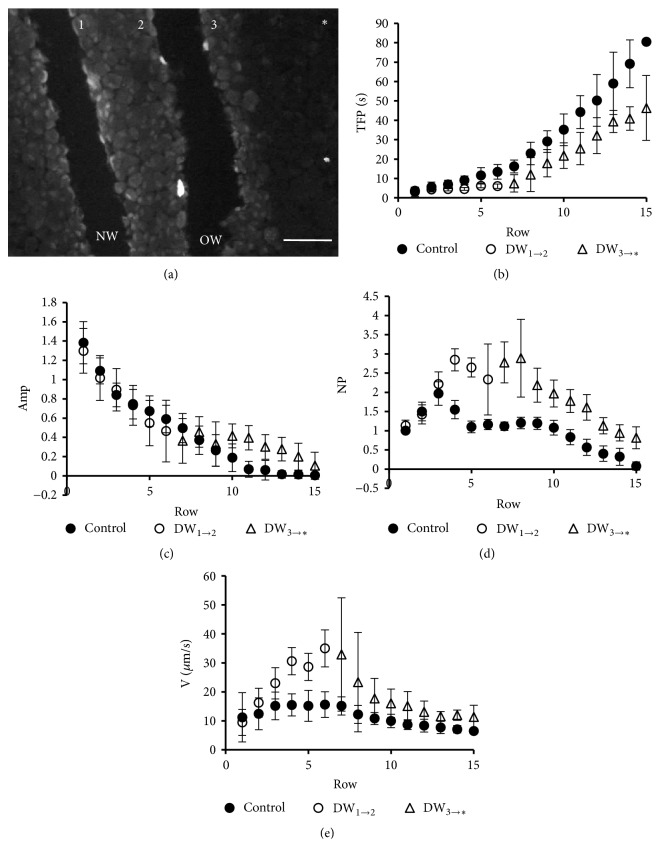
*Average row parameters in double-wounding experiments*. For the double-wounding experiments (a), a first wound (old wound, OW) was performed. A second wound (new wound, NW) was made 10 to 20 min later, to assure complete extinction of the first FCW. In the figure, 1, 2, and 3 designate the wound borders considered for the measurements. The asterisk (*∗*) indicates the direction of the wave to the uninjured tissue. The plots of TFP (b), Amp (c), NP (d), and V (e) vs the row number show that the curves maintain their characteristics beyond the OW, revealing that the tissue gap has not precluded the propagation and properties of the wave triggered by NW. DW_1→2_, and DW_3→*∗*_: fast calcium waves triggered by NW between borders 1 and 2 and between border 3 and the uninjured tissue (**∗**), respectively. Control parameter values were determined in an independent coverslip. Bar = 200 *μ*m. The figure shows results obtained from typical experiments with total numbers of analyzed cells equal to 465 (control) and 425 (double wounding experiment).

Sammak and coworkers [11] demonstrated that a wound-triggered FCW is able to surpass a previously existing discontinuity in an epithelial monolayer. To evaluate if the signal that provokes calcium oscillations during the FCW is transmitted by the extracellular medium, we performed double-wounding experiments (see Materials and Methods; also Supplementary Material, movie 2). As shown in Figure 3(a), the second wound (new wound, NW) was made three to six rows of cells away from the first wound (old wound, OW), which has a width of three to five cell diameters. The second wounds were made slightly oblique to the first ones to allow that all the cell rows (as defined in Materials and Methods) are present in the image field. Figures 3(b)-3(c) shows that some of the parameter values of the calcium wave obtained for the second wound in the double-wounding experiments exhibit profiles similar to the controls. Interestingly, the oscillatory responses are also conserved, although with a somewhat different pattern to the control (Figure 3(d)). Similar results were obtained for the wave velocity (Figure 3(e)). The results shown in Figure 3 suggest that, for the case of BCE cells, the wound-induced calcium wave ultimately depends on an initial diffusive signal (e.g., ATP) and that this signal is per se capable to transmit information to produce calcium oscillations.

**Figure 4:**
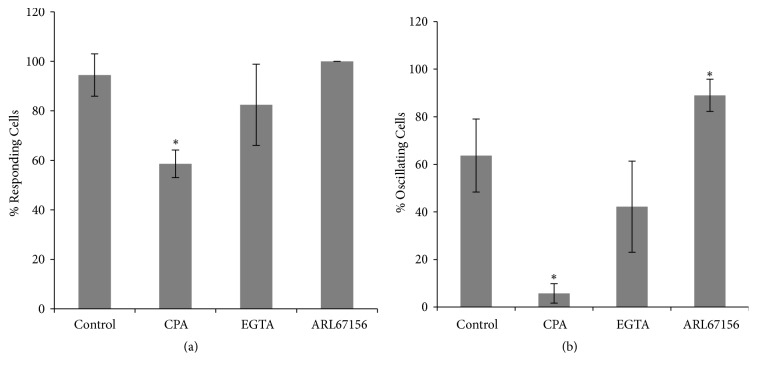
*Percentage of cells exhibiting calcium increase and calcium oscillations during the FCW under different experimental conditions*: (a) Percentage of cells displaying increased cytosolic calcium with respect to the total number of cells in the microscopic field. (b) Percentage of cells displaying calcium oscillations with respect to the total number of cells showing calcium increase. Data correspond to the cells between the 1st and the 15th rows. The asterisk (*∗*) indicates significant difference with respect to control (p < 0.05).

**Figure 5:**
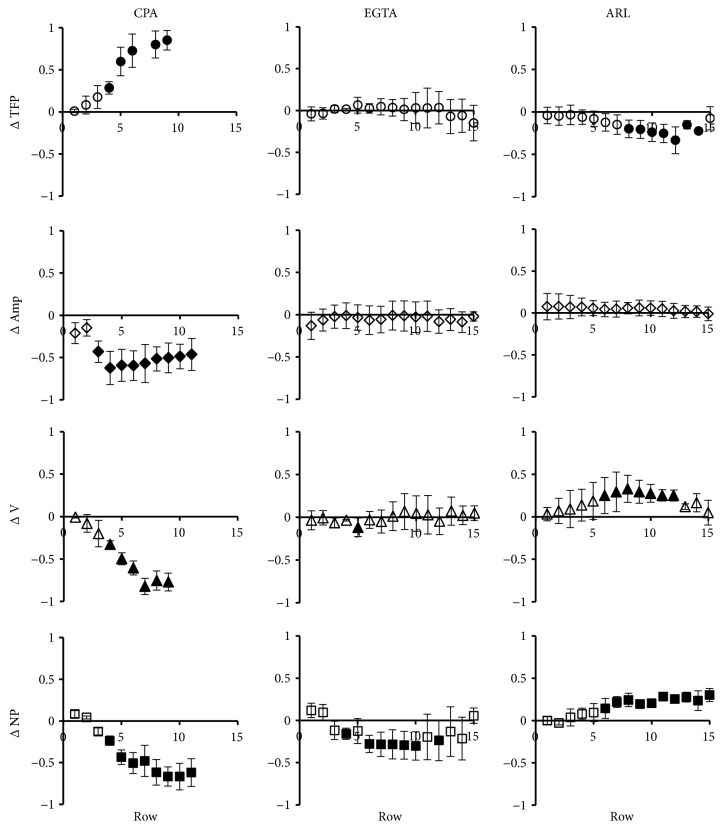
*Effect of CPA, EGTA, and ARL on the dynamic calcium responses*. The figure shows the effects of the Incorporation of 10 *μ*M CPA (left column), 100 *μ*M EGTA (middle column), or 50 *μ*M ARL (right column) on TFP (○), Amp (*◇*), V (△), and NP (□). For these parameters, the plots display the average differences between the treatments and the corresponding controls (∆TFP, ∆Amp, ∆V, and ∆NP) and their standard deviations (for each determination n = 3). Within each experiment all of the values were normalized with respect to the highest one. Filled symbols represent values with significant difference with respect to control (p<0.05). The average total number of analyzed cells was: 418 for CPA, 459 for EGTA, and 701 for ARL.

In previous work we showed that, in wounded BCE cells in culture, the FCW can be abolished by pretreating the cells with a combination of CPA, a reversible inhibitor of the sarcoplasmic/endoplasmic reticulum Ca^2+^-ATPase (SERCA), and EGTA, an extracellular calcium chelator [7, 16]. In order to further understand the roles of extracellular and intracellular calcium in the generation of the cellular calcium signals during the FCW, we analyzed the calcium dynamics in the presence of each one of these chemicals (Figures 4 and 5).  Figure 4 shows the total percentage of cells that increase their cytosolic calcium and the percentage of calcium oscillating cells, and Figure 5 shows the differences in TFP, Amp, V, and NP between CPA and EGTA-treated monolayers with respect to control.

In Figure 4 it can be observed that approximately 59% of the cells of CPA-treated monolayers exhibited a calcium response, with about 10% of them showing oscillatory behavior (around 6% of the total number of cells). These values are significantly different with respect to control. As shown in Figure 5, the drug determines a decrease in the signal amplitude and number of peaks and velocity and provokes a delay in the onset of the first peak. From the 4th row on, all of these parameters become significantly different with respect to control. To be noted, the cells from the tenth row on become unresponsive to CPA treatment.

For the case of EGTA-treated monolayers, about 82% of the cells increased their cytosolic calcium in response to the wound (Figure 4). Out of these, 42% showed oscillatory behavior (34% of the total number of cells). No significant differences were found with respect to control. However, as shown in Figure 5, middle column, EGTA determines a significant reduction in the number of peaks from the 4th to the 11th rows, approximately, without changing the TFP, Amp and V. This is consistent with previous work performed in rat aortic endothelium in situ that suggests that oscillatory changes of calcium concentration during the FCW depend on extracellular calcium influx [56, 57].

**Figure 6:**
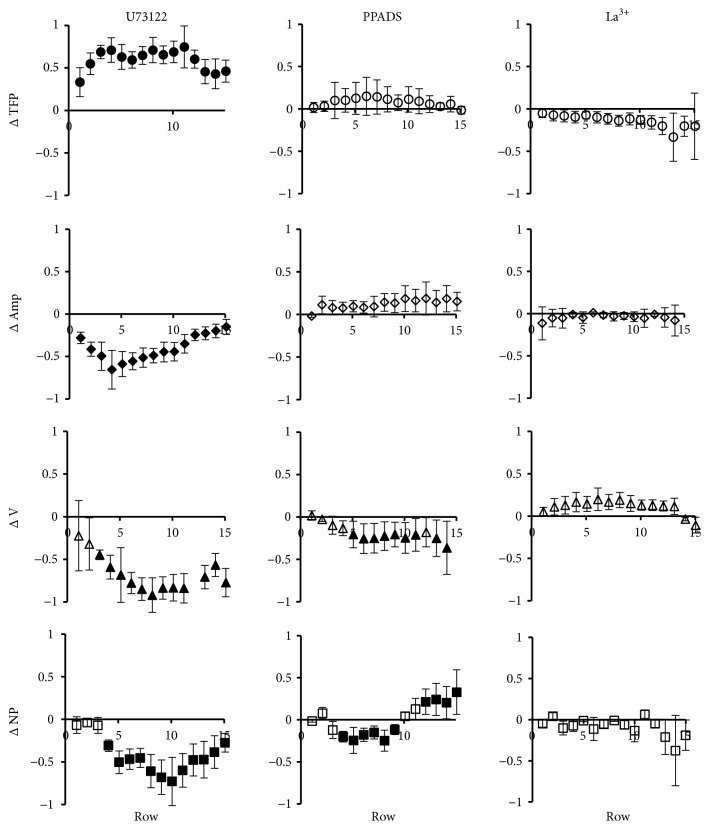
*Effect of U73122, PPADS and La* ^*3+*^ *on the dynamic calcium responses*. Similar to Figure 5, but for experimental media containing 50 *μ*M U73122, 10 *μ*M PPADS, or 10 *μ*M LaCl_3_ (La^3+^). The average total number of analyzed cells was 735 for U73122, 533 for PPADS, and 636 for LaCl_3_.

To further explore the possible molecular mechanisms involved in the calcium increase during the FCW we performed similar studies to Figure 5 but employing U73122, PPADS, and La^3+^ (Figure 6). As mentioned above, the results shown in Figures 3(c)–3(e) suggest that the IP3 signal could participate in the calcium increase during the FCW. Figure 6 shows that incorporation of U73122, an inhibitor of phospholipase C [58] and hence of IP3 production [59], indeed determined analogous effects to CPA on the characteristics of the FCW (see Figure 5). This suggests that as in other cell systems [14, 38, 39, 60], intercellular calcium propagation in the FCW depends, at least partially, on IP3 signaling. The purinergic receptor antagonist PPADS was added at a concentration of 10 *μ*M, far below the ones needed to produce ecto-ATPase inhibition [61]. As can be observed in Figure 6, the drug did not produce significant effects on TFP or Amp but did decrease the wave velocity and the number of peaks up to the tenth cell row. Since PPADS is particularly effective on P2X_1,2,3,5_ [62] and P2Y_1,6,13_ [63], these results suggest that one or more of these specific receptors could also be involved in the oscillatory response. An increase in the number of peaks of the calcium oscillations was observed beyond the tenth row, in principle an effect of complex interpretation, but that could find an explanation in relation with the decrease of ATP with distance. Finally, performance of the wounding experiments in the presence of 10 *μ*M La^3+^, an agent known to block Store Operated Calcium Entry (SOCE) with high sensitivity [64] and also connexin hemichannels [65] at higher concentrations, did not elicit modifications in the wave properties. Considering the lanthanum concentrations employed in this study, our results suggest that SOCEs are not significantly involved in the calcium response of healing BCE cells.

In summary, Figure 5 reveals that CPA determined a decrease in the signal amplitude. In addition, both CPA and EGTA decreased NP, more evidently for the case of CPA. The latter drug also reduced the wave velocity (V). Taken together, the results shown in Figures 4 and 5 suggest a preeminent contribution of intracellular calcium stores in the generation of the FCW, similarly to that observed in other tissues [14, 66–68]. Regarding mechanisms, the main results shown in Figure 6 are that mediation of IP3 seems to participate in determining the characteristics and propagation of the FCW and that some specific purinergic receptors may also be involved.

### 3.2. ATP and Calcium Dynamics

Several works suggest that calcium waves during injury or mechanical stimulation propagate by diffusion of soluble factors released into extracellular medium or via cell to cell through gap junctions [37, 69]. In wing imaginal discs of* Drosophila* larvae, mechanically induced calcium waves depend on cellular prestress and occur via intercellular gap propagation of calcium-induced-calcium-increase signals [70]. As commented above, in many cells types, including corneal endothelial cells, ATP is released during injury and participates in the FCW propagation [22–24]. For the case of BCE cells, we have provided with evidence that the onset and propagation of the FCW depend on extracellular ATP [7], similarly to what was found for other wounded epithelia [14, 66]. In this work we studied the effect of ARL, a weak competitive inhibitor of ecto-ATPases [71], on the characteristics of the calcium response (Figures 4 and 5, right column; also Supplementary Material, movie 3). In uninjured monolayers, ARL has been shown to produce enhancement of the calcium wave propagation in mechanically stimulated BCE cells [72, 73] and increase in oscillatory behavior in MDCK cells [36]. In our system, incorporation of ARL also determined a decrease of TFP and hence an increase in the wave velocity (V). Also, the drug produced a significant increase in the number of peaks (NP) beyond the 5th row, to an extent that becomes larger with the row number, without significant modifications in the amplitude (Figure 5). As shown in Figure 4, in BCE monolayers treated with ARL, 100% of the cells in the field responded to the wound with a cytosolic calcium increase; out of them 92% presented oscillations with significant difference with respect of the control.

**Figure 7:**
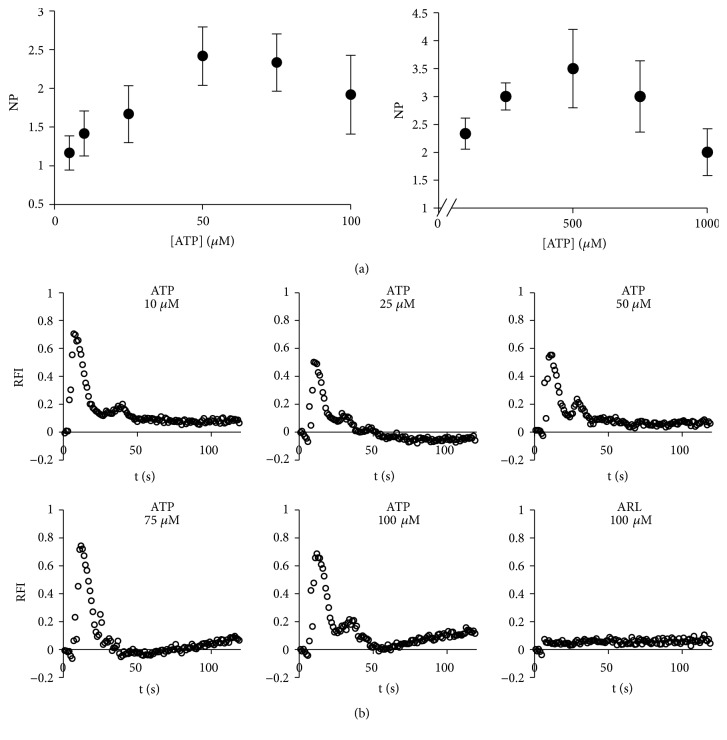
*Oscillatory behavior of cytosolic calcium concentration in uninjured BCE monolayers treated with ATP*. The figure shows the average NP determined for BCE monolayers in the presence of different ATP concentrations (n=3) and single cell recordings of calcium dynamics for the cases of cells representative of the average behaviors at the corresponding ATP concentrations. Also, the figure includes a representative result of calcium dynamics in the presence of 100 *μ*M ARL-67156.

The above results are in agreement with the finding that the inhibitory effect of ARL on the ecto-ATPases is negligible at high ATP concentrations [71]. If, in our system, ATP is released mostly by the cells that died as a consequence of the injury and diffuses along the rest of the monolayer, a higher ATP concentration is expected at the border and immediately neighboring cells. Therefore, the effect of ARL on the cells of the first rows is expected to be very small and would become larger with increasing distance from the wound border. At intermediate ATP concentrations, ARL would be an effective inhibitor of the ecto-ATPases and determine a larger persistence of the ATP signal than the control experiments. Correspondingly, the maintained presence of ATP could be involved in the calcium oscillations. To further explore the plausibility of this hypothesis, we performed studies of the dependence of the number of calcium peaks with ATP (Figure 7) in experiments of ATP-induced calcium waves in uninjured BCE monolayers. As shown in Figure 7(a), we found that, in the interval 0–100 *μ*M, the curve displays a maximum between 50 and 80 *μ*M ATP and is asymmetric, with a lower absolute value for the slope at higher ATP concentrations. These results are consistent with the hypothesis posed above and with evidence regarding the role of ATP in the generation of the calcium oscillations [36, 46–49, 52]. At higher, nonphysiological, ATP concentrations (i.e., 100 to 1000 *μ*M) an increase in the frequency of calcium oscillations can also be observed, yielding an overall bimodal response. A possible interpretation of this latter finding could be that such high ATP concentrations could produce complete saturation of the ecto-ATPases, thus determining a situation analogous to the incorporation of ARL.  Figure 7(b) shows single cell recordings of calcium dynamics in the presence of different ATP concentrations, for the cases of cells representative of the average behaviors at the corresponding ATP concentrations. Also, the figure includes a representative example of cellular calcium dynamics in the presence of 100 *μ*M ARL.

### 3.3. Role of Calcium Oscillations in Apoptosis of Healing BCE Cells

To assess the role of calcium oscillations, ATP scavengers, such as apyrase, have been employed [36]. This approach was not feasible in our case, since apyrase produces an overall abolition of the calcium wave [7]. In the preceding sections we showed that the main effects of EGTA and ARL on the healing BCE cells are to decrease and increase the number of peaks, respectively, without significantly affecting the wave amplitude (Figure 5). For these reasons, we employed ARL and EGTA to provoke modifications in the calcium oscillations and to study their effects on the apoptotic rates of healing BCE cells. CPA was not considered for this study since, although it effectively decreased the number of peaks exhibited by oscillatory cells, it also significantly affected all of the other parameters (Figure 5).

**Figure 8:**
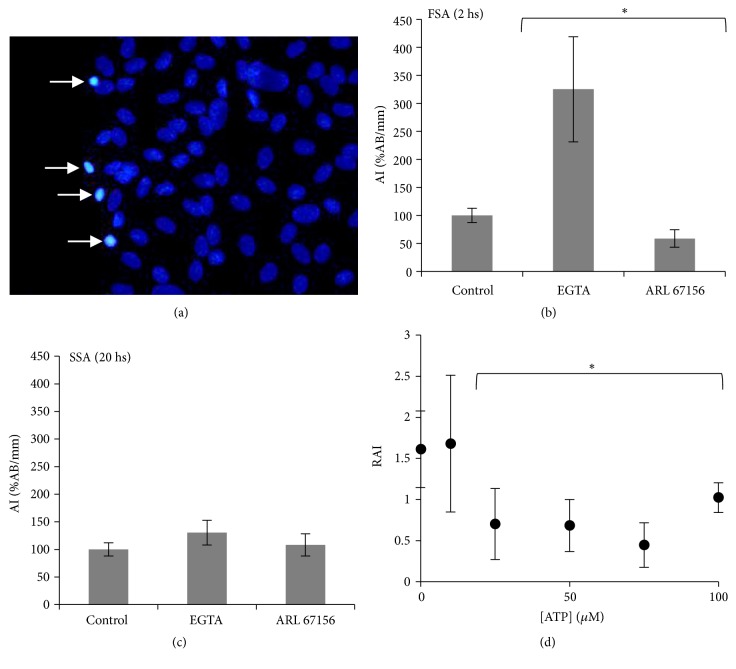
*Effect of EGTA, ARL, and ATP on the apoptotic response during wound healing*. (a) A Hoechst-stained micrograph of a wounded BCE monolayer after 8 hours of healing. The arrows indicate apoptotic bodies at the wound border revealed by intense staining due to nuclei compaction. (b) 2 h after wounding, during the first stage of apoptosis (FSA), incorporation of EGTA determined an approximately 200% increase in the apoptotic index AI (defined as the number of apoptotic bodies AB per millimeter of wound edge, AB/mm). On the contrary, ARL produced a decrease of about 50%. (c) 20 h after wounding, during the second stage of apoptosis (SSA), none of the treatments provoked a significant effect. (d) BCE monolayers were incubated with different ATP concentrations and treated with CPA and EGTA to completely abolish the FCW (see Materials and methods for details). RAI is the corresponding apoptotic index relative to control. The asterisk (*∗*) indicates significant difference with respect to control (p < 0.05).

In previous work we showed that, during wound healing, BCE monolayers exhibit a high apoptotic rate of the front cells and that this apoptotic response takes place in two well-defined stages [7]. The first stage reaches its maximum at two hours of healing, while the second one starts at about eight hours after the injury and increases linearly with the migrated distance. Hence, for this work we studied the effect of ARL and EGTA on the apoptotic rate at times characteristic of each stage, 2 and 20 hours after wounding.  Figure 8 reveals that while EGTA determines a great increase in the apoptotic index of the first stage, ARL provokes a significant reduction (Figure 8(b)). Neither drug affects the apoptotic rate of the second stage (Figure 8(c)).

Although as shown (Figure 5, right column) ARL also decreases the TFP and hence increases the velocity of propagation of the wave, we interpreted its effects on apoptosis as a result of the increase in calcium oscillations, in view of previous evidence reported for uninjured tissues [44, 45]. To obtain further support for this hypothesis, we performed experiments of wound healing in conditions of complete FCW inhibition (i.e., employing a combination of CPA and EGTA, see Materials and Methods), in order to maintain the first stage of apoptosis and to exclude the influence of the calcium signals. In these experiments, 30 min before the wound, just before the CPA incubation, we preincubated the cells with 10 to 100 *μ*M ATP for 2 min. In this way we were able to induce ATP-dependent calcium responses characterized by different numbers of peaks, in the absence of wound-dependent calcium waves that would propagate at a certain velocity.  Figure 8(d) shows the apoptotic indexes relative to controls (RAI), as functions of the exogenous ATP concentration, obtained 2 h after wounding (i.e., first stage of apoptosis). Under these conditions, RAI decreases with the ATP concentration to a minimum of approximately 75 *μ*M, a value in the range of the ATP concentrations that induce the maximum number of peaks (cf. Figure 7). The results shown in Figure 8 suggest that ATP-induced calcium oscillations during the FCW could participate in the modulation of the apoptotic response of healing BCE cells during the first stage of apoptosis.

## 4. Discussion

In response to a mechanical injury, confluent cultures of bovine corneal endothelial cells develop a calcium wave that propagates from the wound border to the rest of the monolayer and lasts for 3 to 5 min. During this wave, some cells exhibit oscillations in cytosolic calcium. The objectives of this work have been to characterize the cell calcium dynamics during the FCW and to explore the possible role of the calcium oscillations in the healing process. At least for the case of BCE cells, it is methodologically very difficult to specifically modify calcium oscillations without affecting other properties of the overall calcium wave. Ideally, the most conclusive way to study the effect of calcium oscillations would be to completely inhibit them without affecting the other properties of the FCW. ATP scavengers, such as apyrase, or the SERCA inhibitor CPA, decrease calcium oscillations but also the amplitude of the calcium signal [36]. In particular, in wounded BCE monolayers incorporation of apyrase almost completely abolishes the calcium response [7]. We have instead utilized EGTA, an extracellular calcium chelator that in BCE cells significantly decreases calcium oscillations, and ARL, a competitive inhibitor of ecto-ATPases, that determines significant increases in the oscillatory response and also affects the wave velocity. The two drugs do not affect other wave properties.

In the first part of this work we made a characterization of the cellular calcium response during the FCW and the basic mechanisms involved. In particular, this characterization allowed us to select the appropriate drugs to modify calcium oscillations and study the effects on the apoptotic responses of healing BCE monolayers. For this, our approach consisted in a thorough study of the calcium dynamics of each cell in the microscopic field during the FCW and a classification of the cells in consecutive rows starting from the wound border. The results were represented as the average of the cellular responses per row as functions of the row number. The double-wounding experiments showed that the wave maintains its general characteristics beyond a preexisting gap, suggesting that in BCE cells an extracellular diffusive signal constitutes the main mechanism for its propagation, possibly in a “Release and Diffusion” manner [68]. A large amount of evidence suggests that, in most cell types [13, 14, 23, 68, 74], including BCE cells [7], ATP released from damaged and stressed cells at the wound borders represents the main contributor to this diffusive signal. In addition, evidence from this work showed that in BCE cells calcium from intracellular stores represents a major source for the cytosolic calcium rise during the FCW. Our results also suggest that similarly to other systems, IP3 participates in the propagation of the calcium wave and in the generation of calcium oscillations.

In this work we show that, in monolayers of BCE cells in culture, wounds performed in calcium-free solutions with EGTA elicit an FCW with a significant decrease in the number of calcium peaks, without affecting other parameters of the wave. This finding is consistent with work on endothelium of rat aorta in situ that suggests that the oscillatory behavior of the cells is determined by extracellular calcium influx [56]. The authors also obtained results suggesting that during the FCW the connexin hemichannels are opened, provoking an inward sodium current that induces the reverse mode of operation of the sodium-calcium exchanger, with the consequent calcium influx responsible for the oscillations [57]. Our results do not show evidence of intracellular sodium changes during the FCW (data not shown), suggesting that in BCE cells the mechanism of extracellular calcium entry is different. Taking into account that the purinergic receptors P2X are nonselective cation-conducting channels or are associated with hemichannels in a modulatory way [75, 76], it is possible that in BCE cells the oscillatory behavior during the FCW at least partially depends on the activation of P2X receptors that ultimately allow extracellular calcium influx in a sodium-independent way. This view is consistent with our results concerning the participation of ATP in the calcium responses [7] and with those obtained in this study from employment of PPADS, a blocker of purinergic receptors that suggest that the response could involve receptors P2X_1,2,3,5_ and P2Y_1,6,13_. We did not obtain evidence that other sources of calcium, such as those mediated by SOCEs, could contribute to the generation of the calcium wave in healing BCE cells. In our system, EGTA would decrease calcium oscillations by approximately setting extracellular free calcium concentration to zero and thus preventing calcium influx. As shown in this study, EGTA determined a large increase in the apoptotic index of the first stage of apoptosis, without affecting the apoptotic response during the second stage.

Contrary to EGTA, the most important effect of ARL on the healing process of BCE monolayers was to produce a significant decrease (i.e., to about half the control value) in the apoptotic index of the first stage of apoptosis, also without affecting the second apoptotic stage. We have interpreted the ultimate effect of ARL on apoptosis as due to the maintenance of moderately risen ATP levels during the FCW. ARL has been found to inhibit ecto-ATPase-dependent induction of neutrophil apoptosis by T_REG_ lymphocytes [77]. We are proposing here that the drug affects apoptosis via the enhancement of cytosolic calcium oscillations during the FCW. In uninjured tissues, calcium oscillations have been reported to determine antiapoptotic responses. Thus, in T-lymphocytes, stimulus-induced calcium oscillations promote cell survival and proliferation via a mechanism that involves the transcription factor NFAT and increased expression of II-2 [45, 78]. In mouse oocytes, oscillations in cytosolic calcium determine survival or apoptotic responses, depending on the oocyte age [43, 44]. In COS-7 cells, the antiapoptotic protein Bcl-2 has been reported to enhance IP3R-mediated calcium oscillations and thus promote cell survival [79].

In this study, the effects of ARL and EGTA on the apoptotic rate of the first stage of apoptosis have been therefore interpreted on the basis of their effects on different properties of the dynamics of the cellular calcium increase during the FCW. We have observed that neither drug significantly modifies the total percentage of cells that exhibit increased calcium. In addition, incorporation of EGTA does not provoke changes on the percentage of cells displaying oscillatory behavior. However, the results are suggestive that EGTA could participate in the apoptotic response via a decrease in the calcium oscillatory frequency, while ARL would have the opposite effect on apoptosis by increasing that frequency. For the case of ARL it cannot be excluded the possibility that the drug could also participate by increasing the percentage of cells displaying calcium oscillations during the FCW.

## 5. Conclusions

The results of this work show that during the FCW triggered by a mechanical injury of BCE monolayers many cells exhibit oscillatory behavior in response, at least partially, to a diffusive signal, possibly ATP. Manipulation of this behavior with EGTA and ARL shows that the drugs have opposite effects on the oscillatory responses and on the apoptotic index of the first stage of apoptosis. Hence, the results presented here suggest a possible role for the calcium oscillations exhibited by BCE cells in culture during the healing response to mechanical injury, namely, to modulate the apoptotic response of the first stage of apoptosis. Since both stages are caspase-dependent [7], similar basic pathways could a priori be expected to trigger the apoptotic response. The fact that, during wound healing of BCE cells, only the first stage of apoptosis is affected by EGTA and ARL is therefore noteworthy and suggests that the two stages are at least partially subject to independent regulation.

## Data Availability

The data used to support the findings of this study are available from the corresponding author upon request.

## Abbreviations

AB: Apoptotic bodies
AI: Apoptotic index
Amp: Amplitude of the first peak
ARL: ARL 67156
BCE: Bovine corneal endothelial
CPA: Cyclopiazonic acid
CS: Control solution
FCW: Fast calcium wave
FSA: First stage of apoptosis
NP: Number of identifiable peaks
NW: New wound
OW: Old wound
PPADS: Pyridoxalphosphate-6-azophenyl-2′,4′-disulphonic acid
SSA: Second stage of apoptosis
TFP: Time to reach the first peak after injury
V: Calcium wave velocity.

## Supplementary Materials

Supplementary Materials Movie 1: FCW in control conditions, 1 frame per second, duration 2 min. Movie 2: FCW in double wounding experiments, 1 frame per second, duration 2 min. Movie 3: Effect of ARL on FCW, 1 frame per second, duration 2 min.
